# Genome Sequences of Green- and Brown-Colored Strains of Chlorobium phaeovibrioides with Gas Vesicles

**DOI:** 10.1128/MRA.00711-19

**Published:** 2019-07-18

**Authors:** Denis S. Grouzdev, Olga N. Lunina, Vasil A. Gaisin, Maria S. Krutkina, Roman V. Baslerov, Alexander S. Savvichev, Vladimir M. Gorlenko

**Affiliations:** a Research Center of Biotechnology, Russian Academy of Sciences, Moscow, Russia; University of Maryland School of Medicine

## Abstract

The draft genomes of green-colored Chlorobium phaeovibrioides *Gr*Khr17 and brown-colored Chlorobium phaeovibrioides *Br*Khr17, green sulfur bacteria with gas vesicles isolated from Lake Bolshye Khruslomeny, are presented. These sequences contribute to genomic analyses of the *Chlorobiaceae* family that are part of ongoing research seeking to better understand their ecosystem-specific adaptations.

## ANNOUNCEMENT

**FIG 1:**
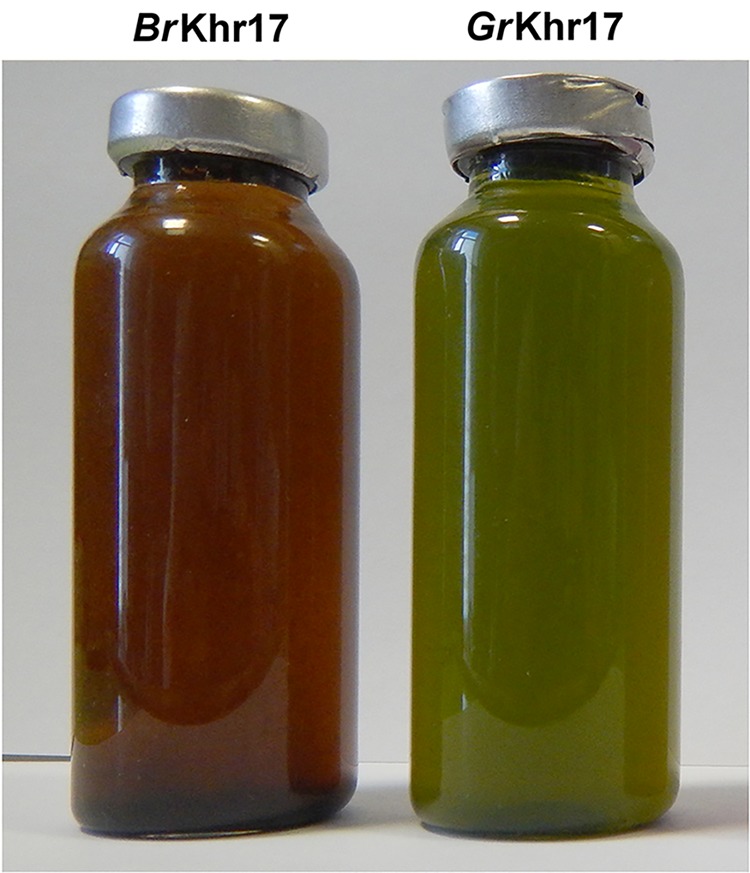
Color of the cultures of Chlorobium phaeovibrioides strains *Br*Khr17 and *Gr*Khr17.

Meromictic lakes along the White Sea region harbor many populations of green sulfur bacteria (GSB) that offer a great opportunity for study diversity in examining the evolution of green- and brown-colored types of GSB (1–4). The green-colored strain *Gr*Khr17 and brown-colored strain *Gr*Khr17 of GSB (Fig. 1) were isolated from the chemocline of the meromictic Lake Bolshye Khruslomeny (Oleniy Island, Kovda Inlet, Kandalaksha Gulf, White Sea). The strains were maintained using a recently described medium (5) with sodium bicarbonate (1.5 g liter^−1^) and sodium sulfide (0.5 g liter^−1^) at 20 to 25°C in light (2,000 lx) under anaerobic conditions. The main pigment of strain *Gr*Khr17 was bacteriochlorophyll (BChl) *d*, and the main pigment of strain *Br*Khr17 was BChl *e.* The pigments were determined in a 50% glycerol cell suspension using a spectrophotometer (SF-56A, OKB Spectr). A particular feature of the strains was the presence of gas vesicles, which are absent in Chlorobium phaeovibrioides DSM 269^T^ and Chlorobium phaeovibrioides DSM 265 (6, 7).

DNA was purified from the bacterial colonies grown in the semisolid medium described earlier, and under the same conditions, using the cetyl trimethylammonium bromide (CTAB) method (8). A NEBNext Ultra DNA library prep kit (New England Biolabs, USA) was used to prepare fragment libraries for genome sequencing. Sequencing was undertaken using the Illumina HiSeq 1500 platform with single-end 250-bp reads. A total of 404,903 and 432,293 reads were obtained from *Gr*Khr17 and *Br*Khr17, respectively. Low-quality reads were trimmed using a Trimmomatic v. 0.36 (9) with default settings. Subsequently, the quality-filtered reads were *de novo* assembled with SPAdes v. 3.12.0 using default settings (10). The final draft genome assembly of *Gr*Khr17 contained 40 scaffolds, covering a total of 1,959,778 bp, with an *L*_50_/*N*_50_ value of 4/187,559, a G+C content of 52.73%, and an average sequence coverage of 33×. The final draft genome assembly of *Br*Khr17 contained 67 scaffolds, covering a total of 2,094,018 bp, with an *L*_50_/*N*_50_ value of 5/161,558, a G+C content of 53.04%, and an average sequence coverage of 31×. Identification of the protein-coding sequences and primary annotation were performed using the NCBI Prokaryotic Genome Annotation Pipeline (PGAP v. 4.7) (11), which identified 1,910 genes, 1,817 protein-coding sequences, 46 pseudogenes, and 51 RNA genes for strain *Gr*Khr17, and 1,994 genes, 1,876 protein-coding sequences, 67 pseudogenes, and 51 RNA genes for strain *Br*Khr17. Functional annotation of the protein-coding genes was performed using BlastKOALA (12) and supported with BLASTp (E value < 1e-20) (13) searches against the NCBI nonredundant protein database.

The 16S rRNA sequence analysis using the nucleotide BLAST (13) revealed that *Gr*Khr17 and *Br*Khr17 share 99.93% and 99.87% similarity, respectively, with Chlorobium phaeovibrioides DSM 265 (GenBank accession number CP000607). The average nucleotide identity (ANI) and digital DNA-DNA hybridization (dDDH) values were calculated using the ANI calculator from the Kostas lab (http://enve-omics.ce.gatech.edu/ani) (14) and the Genome-to-Genome Distance Calculator (GGDC) v. 2.1 (http://ggdc.dsmz.de/ggdc.php) (15), respectively. In comparison with Chlorobium phaeovibrioides DSM 265, the ANI values of strains *Gr*Khr17 and *Br*Khr17 were 99.1% and 99.0%, and the dDDH values were 91.7% and 90.6%, respectively. The calculated values exceeded the proposed species boundary values for species delineation (ANI < 95 to 96%, dDDH < 70%) (16), which suggests that strains *Gr*Khr17 and *Br*Khr17 are novel strains of the known species Chlorobium phaeovibrioides.

The genomes of Chlorobium phaeovibrioides *Gr*Khr17 and *Br*Khr17 contain the *gvp* gene cluster (17), which encodes proteins that are involved in gas vesicle biogenesis (18). The genome of Chlorobium phaeovibrioides *Br*Khr17 contains the BChl *e* gene cluster (19), which includes BChl *e* biosynthesis genes *bciD* and *bchF3*, as well as the *cruB* gene, which is responsible for the biosynthesis of isorenieratene (20). Chlorobium phaeovibrioides *Gr*Khr17 does not possess the BChl *e* gene cluster. Both announced genomes lack genes of the *sox* system for thiosulfate oxidation. Sequencing and analysis of these bacteria revealed genomic determinants of the particular phenotype of new strains of Chlorobium phaeovibrioides from Arctic meromictic lakes.

### Data availability.

These whole-genome projects have been deposited in DDBJ/ENA/GenBank under the accession numbers RXYJ00000000 (Chlorobium phaeovibrioides *Gr*Khr17) and RXYK00000000 (Chlorobium phaeovibrioides *Br*Khr17). The versions described in this paper are RXYJ01000000 and RXYK01000000, respectively. Raw sequence reads are available under the SRA accession numbers SRR9141222 (Chlorobium phaeovibrioides *Gr*Khr17) and SRR9141223 (Chlorobium phaeovibrioides *Br*Khr17).
